# Crystal Structure of Major Envelope Protein VP24 from White Spot Syndrome Virus

**DOI:** 10.1038/srep32309

**Published:** 2016-08-30

**Authors:** Lifang Sun, Yintao Su, Yanhe Zhao, Zheng-qing Fu, Yunkun Wu

**Affiliations:** 1 State Key Laboratory of Structural Chemistry, Fujian Institute of Research on the Structure of Matter, Chinese Academy of Sciences, Fuzhou 350002, China; 2 Department of Biochemistry and Molecular Biology, University of Georgia, Athens, GA 30602, USA

## Abstract

White spot syndrome virus (WSSV) is one of the major and most serious pathogen in the shrimp industry. As one of the most abundant envelope protein, VP24 acts as a core protein interacting with other structure proteins and plays an important role in virus assembly and infection. Here, we have presented the crystal structure of VP24 from WSSV. In the structure, VP24 consists of a nine-stranded β–barrel fold with mostly antiparallel β-strands, and the loops extending out the β–barrel at both N-terminus and C-terminus, which is distinct to those of the other two major envelope proteins VP28 and VP26. Structural comparison of VP24 with VP26 and VP28 reveals opposite electrostatic surface potential properties of them. These structural differences could provide insight into their differential functional mechanisms and roles for virus assembly and infection. Moreover, the structure reveals a trimeric assembly, suggesting a likely natural conformation of VP24 in viral envelope. Therefore, in addition to confirming the evolutionary relationship among the three abundant envelope proteins of WSSV, our structural studies also facilitate a better understanding of the molecular mechanism underlying special roles of VP24 in WSSV assembly and infection.

White spot syndrome virus (WSSV) is a rod-shaped enveloped virus with a large, double-stranded DNA genome^1^,^2^,^3^,^4^, and belongs to the family *Nimaviridae* as the sole member of a novel genus *Whispovirus*^5^,^6^. The virus has a wide host range of most species of crustaceans^7^, and therefore becomes the major and most serious pathogen in cultured shrimp and has caused severe mortality and huge economic losses to the world’s shrimp farming industry^8^. So far, three geographic WSSV isolates have been sequenced, and in the complete genome sequence approximately 180 open reading frames are likely to encode functional proteins^5^,^9^, most of which share no homology to any known proteins or motifs^10^. Using proteomic method, more than 50 structural proteins are known, of which 22 are envelope proteins^11^,^12^,^13^. The envelope proteins have absorbed a lot attention due to their critical roles in virus entry, virus assembly, host cell targeting as well as host defense triggering^14^.

It has been indicated that VP28, VP26, VP24, and VP19 are the four most abundant envelope proteins of WSSV^4^,^11^,^15^. Neither of which shares significant homology with known structural proteins from other viruses^5^. However, VP28, VP26 and VP24 share significant sequence similarities. Previous studies have showed that VP28 is crucial for virus entry involving in attaching and penetrating into host cells^16^,^17^, while VP24 could not bind to host cell membrane though it could interact with VP28 to form a protein complex and participate in virus infection together^18^,^19^. Moreover, studies have further shown VP24 could bind with many other structural proteins including VP28, VP26, VP19, VP33, VP38, VP51A, VP53A and wsv010 for its function in WSSV infection or morphogenesis^11^,^20^,^21^,^22^,^23^. For the interaction, VP24 has been recognized as a core protein to associate with other structural protein partners and form an envelope protein complex, serving as a hub protein to function in cell recognition, cell attaching and guidance of virus entry^24^,^25^. Recently, VP24 has been demonstrated to be a chitin-binding protein involved in WSSV infection^26^. However, the exact role and molecular mechanisms of VP24 in WSSV infection and assembly remain elusive.

Identification the proteins involved is crucial to the understanding of the molecular events. The structural interpretation of them will provide valuable information for understanding their acting modes. Till now, only two three-dimensional structures of WSSV structural proteins, VP26 and VP28, were reported to occur as trimers in the viral envelope, further supposed VP26 and VP28 were located on the outer surface of the virus and were observed as a surface protrusion in the envelope by immunoelectron microscopy^27^. A structural elucidation of VP24 was hampered by the failed attempt to obtain soluble VP24 protein full-length or fragments.

Here, we have successfully prepared soluble VP24 protein by adding a non-denaturing protein-solubilizing agent and finally determined the crystal structure of VP24. As one of the major envelope proteins, VP24 forms a trimer with each monomer adopting a typical nine-stranded β-barrel architectures with a conservative hydrophobic core similar to that of VP28 and VP26, but with a totally different N-terminal region and a distinct protruding C-terminus. The electrostatic surface potential calculation of VP24 further reveals almost opposite surface properties of both the front and bottom surfaces to those of VP28 and VP26, which could explain the mechanistic difference in host-cell membrane attachment for VP24 and VP28. In addition to confirming the evolutionary conservation among the three abundant envelope proteins of WSSV, VP24, VP26 and VP28, our structural studies also provide insight into a better understanding of the molecular mechanism underlying special roles of VP24 in WSSV infection and assembly.

## Results and Discussion

### Crystal structure of VP24

The transmembrane truncated VP24 (residues 28-208) was expressed and purified in *E. coli* BL21 (DE3) successfully. However, a lot of precipitation was appeared while the recombinant VP24 concentration reached above 0.8 mg/ml. Lucky, after screening, with the addition of non-denaturing protein-solubilizing agent NDSB-201, soluble VP24 could reach a concentration of 3.5 mg/ml and yield crystals. Using SAD phasing, the crystal structure was determined to a resolution of 2.4 Å with a final R_work_ of 0.17 and R_free_ of 0.19 (refinement statistics see Table 1). The final monomer model of VP24 contains residues 40-208.

**Figure 1:**
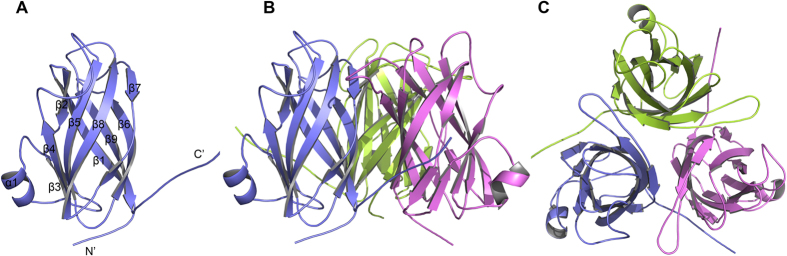
Structure of VP24. (**A**) Cartoon diagram of the VP24 monomer. The secondary structural elements, N and C termini are labeled. (**B**) Cartoon diagram of the VP24 trimer (crystallographic symmetry-related molecules). (**C**) Top view of the VP24 trimer. Diagrams were prepared using the program PyMol.

**Figure 2:**
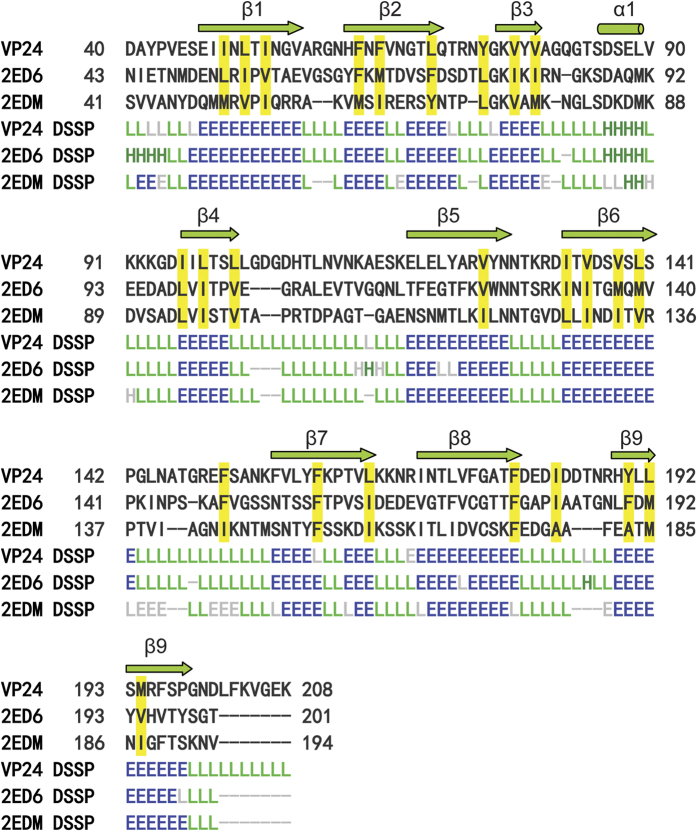
The structure and the sequence alignment of VP24 with VP28 (PDB: 2ED6) and VP26 (PDB: 2EDM). The secondary structure is assigned by DSSP information for helix (H), strand (E) and coil (L), respectively. Residues of hydrophobic side chains lining the inner surface of the β–barrel core are highlighted in yellow boxes. This figure was created by using DaliLite.

The overall structure comprises nine β strands and one α helix, of which the nine antiparallel β-strands adopt a β–barrel fold to form the core of the protein, in which strand 9 is hydrogen-bonded to strand 1, and the short α helix on the turn between β3 and β4 is hanged out the β–barrel fold (Fig. 1A). In the crystal structure, VP24 adopts a nine-stranded β–barrel fold with mostly antiparallel β-strands (Fig. 1A). It’s noted that β2 and β7 both have a kink in strands. Inside the β-barrel, it is a highly hydrophobic core. The inner surface lining of the core is mainly contributed by the hydrophobic side chains from five Ile, seven Leu, five Phe, five Val, two Tyr and one Met residues; the hydrophobicity of the core is quite conserved among VP24, VP28 and VP26 (Fig. 2). The N-terminal and C-terminal loops extend outside the β-barrel to opposite direction, both almost perpendicular to the axis of the β–barrel (Fig. 1).

### Oligomization of VP24

**Figure 3:**
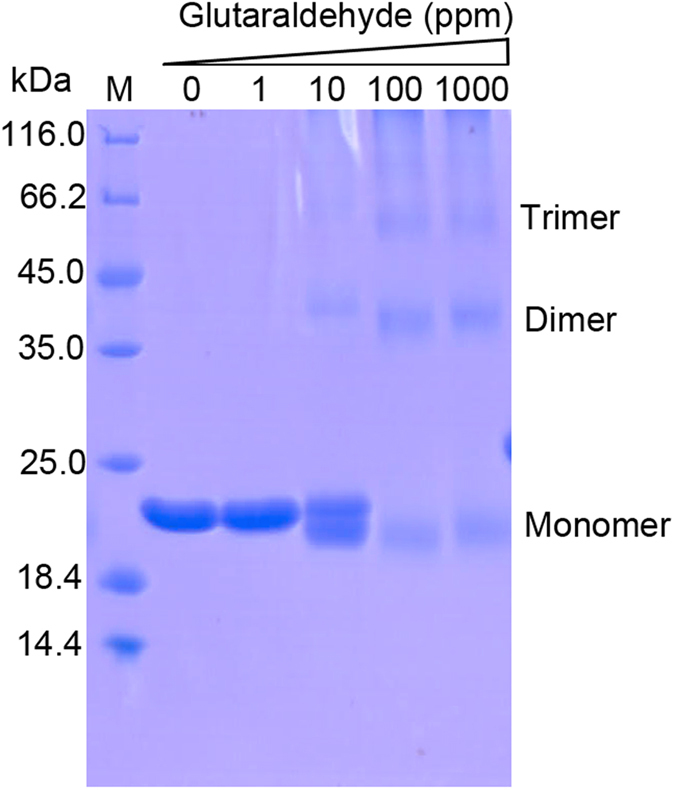
Cross-linking assay of purified VP24. Increasing amounts of glutaraldehyde (0, 1, 10, 100, 1000 ppm) were incubated with the purified VP24. The samples were analyzed by 15% SDS-PAGE. M: protein marker.

**Figure 4:**
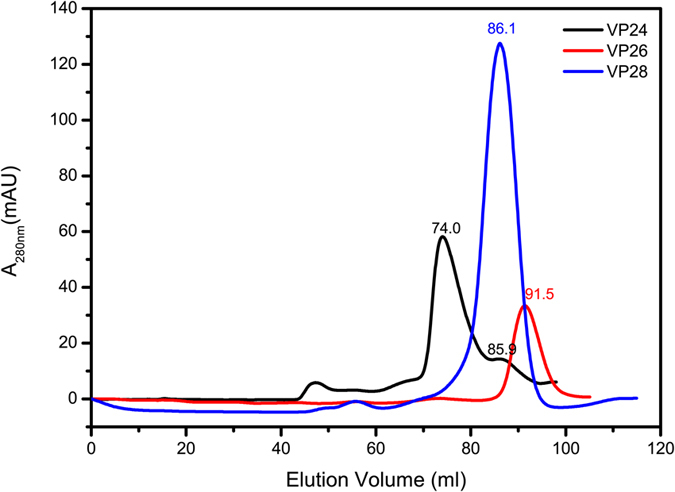
The oligomerization state of recombinant VP24 was analyzed in the gel filtration of Superdex 200 column (GE Healthcare) and compared to those of VP26 and VP28.

In the crystal, VP24 forms a trimer by crystallographic three-fold symmetry axis (Fig. 1). The trimer interface has a buried surface area of 1187.1 Å^2^, accounting for 12.6% of total monomer surface area (http://pdbe.org/pisa/). And the trimer interaction mainly contributed by β strands β1-β9-β6 and β7’. To further validate the trimeric formation, a chemical cross-linking assay indicated that glutaraldehyde cross-linked VP24 into trimers in a concentration-dependent manner, suggesting a trimer formation in solution (Fig. 3). Furthermore, the oligomerization state of recombinant VP24 was also investigated by gel filtration chromatography and comparison with VP26 and VP28. In the gel filtration analysis, VP24 preferred an oligomerization state rather than a monomer state (Fig. 4). Accordingly, in our judgement, the trimeric assembly observed in crystal structures similar to that of VP28 or VP26 should represent a natural conformation in the viral envelope, rather than the dimer formation as previously suggested^24^. However, the trimer interaction is weak, suggesting a possible monomer-trimer transition during WSSV biological process. This is consistent with the previous reports of viral structural proteins exhibiting multiple functions by adopting different oligomerization states^28^,^29^.

### Structural comparison of VP24 with VP28 and VP26

**Figure 5:**
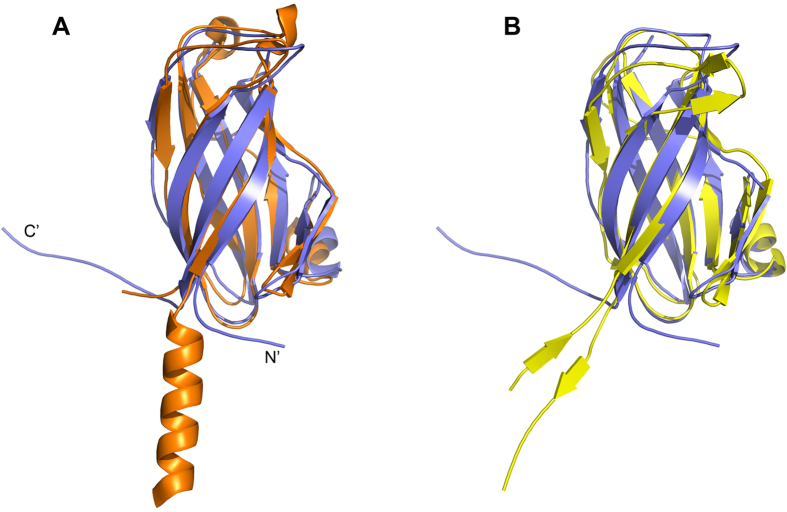
The superposition of VP24 (slate) to VP28 (orange) (A) and VP26 (yellow) (B).

With the sequence similarity shared by VP24 with VP28 and VP26, the VP24 structure can be well superimposed to that of VP28 (PDB: 2ED6) and VP26 (PDB: 2EDM) with a RMSD for backbone atom of 1.9 Å and 2.5 Å, respectively. In overall, the three envelope proteins share a conserved nine-stranded β–barrel fold (Fig. 5), which is different from the predominant eight-stranded β–barrel, a viral canonical jelly-roll fold, commonly observed in other viral proteins^30^,^31^,^32^,^33^,^34^,^35^,^36^,^37^. This structural similarity suggests that the three WSSV envelope proteins could have evolved from a common ancestral origin via gene duplication^38^, such as the three β-jelly roll folds in picorna-like virus capsids that could evolved by triplication of a single domain and further independent evolution of the three domains^39^,^40^.

However, some distinct observes in their structures. Compared to VP28, VP24 lacks the two short 3_10_ helix at the top of the β–barrel, and in contrast to VP26, it did not have a short pair β-sheet at the top of the β–barrel (Fig. 5). Furthermore, significantly different to those of VP26 and VP28, the N-terminus of VP24 forms a loop perpendicular to the barrel axis, which may pull the protein more closely attached to the viral envelope, compared to the protruding helix bundle parallel to the barrel axis of VP28 (Figs 2 and 5). As a linker connecting to transmembrane helix anchor, the N-terminus of VP24, VP26 and VP28 with distinct topology may implicate their differential roles for virus assembly. And the C-terminal of VP24 also extends out the barrel as a flexible long loop, which might grant the C-terminus more dynamics and facilitate VP24 to recruit other structural proteins.

**Figure 6:**
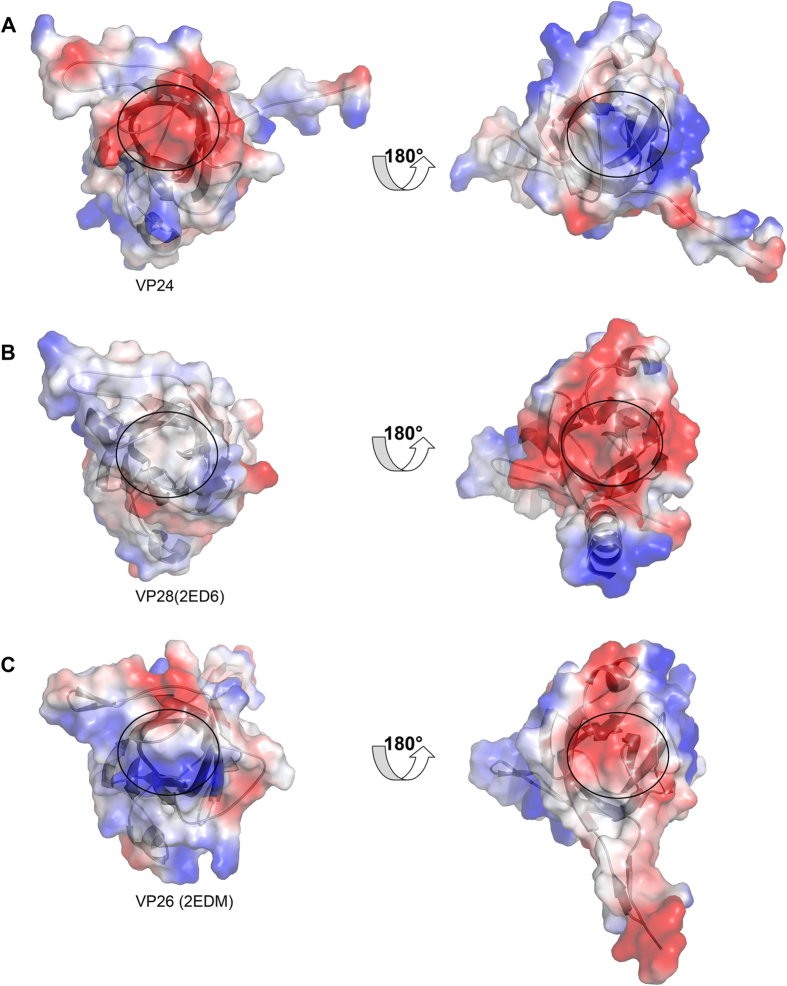
Electrostatic surface potential of VP24 (A), VP28 (B), and VP26 (C). The right line was the structure of their 180 degree rotation view, respectively. The core of the β-barrel was highlighted by black circle.

More interestingly, the electrostatic surface potential measurement of VP24 structure reveals an electronegative surface (Fig. 6A), owing to the presence of residues aspartate and glutamate residues at the top of β–barrel core (away from the viral envelope), and a highly electropositive surface at the bottom of the barrel core, distinct to those of VP28 and VP26 (Fig. 6BC). As shown in Fig. 6, in contrast to VP24, VP28 is electroneutrality and electronegative, respectively; while VP26 almost has the opposite electrostatic surface. This might suggest different preference of ligand recognition of them. Furthermore, VP24 is considered the core of the infectome, these distinct surface features should reasonably explain the mechanism underlying the different binding property of them with other viral proteins or host protein as previously described^18^,^23^,^24^,^25^,^26^, and further provide insight into their different roles in WSSV assembly and infection.

### Interaction analyses of VP24

Previous studies have showed that VP24, VP28, and VP26 could interact with each other and form a complex^11^. To assess binding affinities of VP24 to VP28 and VP26 respectively, *in vitro* ITC assay indicated that the interaction could be too weak to be measured from the heat generated by reaction (Supplementary Figure). However, in the previous report, it has been shown that three C-terminal deleted mutants VP24_26–172_, VP24_26–135_, VP24_26–98_ and three N-terminal deleted mutants VP24_62–208_, VP24_99–208_, VP24_136–208_ could all interact with VP28^41^. It implies the N terminus and C terminus of VP24 both take part in the interaction with VP28. According to the trimer structure of VP24, the oligomer interfaces are composed mainly by strands β1-β9-β6 and β7’, which are included mostly in above-mentioned VP24 mutants. It is thus speculated that major envelope proteins VP24 and VP28 might interact with each other to form a heterotrimer as well as a self-interaction homotrimer because of the similar interfaces. Moreover, a recent report has shown that VP24 is a chitin-binding protein involved into WSSV infection through the chitin-binding domain of VP24 containing residues186-200^26^. In the VP24 structure, this chitin-binding domain including strand β9 located at the interface of the trimer. This further supports that VP24 could function as a monomer to present β9 at the surface for chitin binding during virus infection and undergo a monomer-trimer transition for different biological roles.

In conclusion, we presented a high resolution structure of WSSV major envelope protein VP24 with a unique nine-stranded β–barrel fold with mostly antiparallel β-strands, shared by the two mentioned WSSV envelope proteins but distinct from the featured eight-stranded jelly roll of other viral proteins. The structure also reveals distinct topologies of both the N-terminal and C-terminal loop extending out the β–barrel. Further, the electrostatic surface potential calculation reveals an almost contrary electrostatic property for the front and bottom surfaces of VP24 compared to VP28 and VP26, which could provide mechanistic insight into the distinct function of them for virus infection.

**Figure 7:**
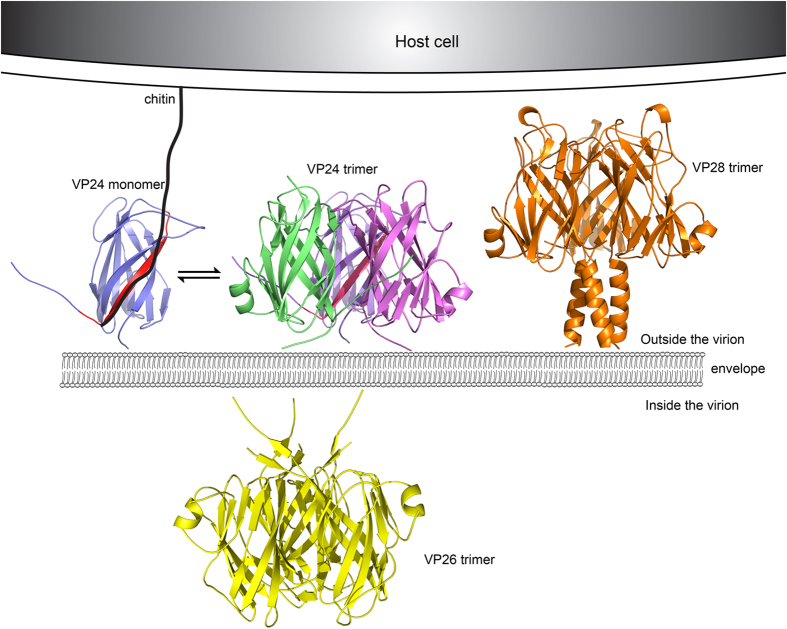
3D models of the location of VP24, VP28 and VP26. VP24 and VP28 anchor on the outside viral envelope membrane, while VP26 anchor on the inside viral envelop membrane. The proteins would have a reversible monomer to trimer transition. VP24 could bind to chitin via its β9 strand (colored by red) which may draw the distance between WSSV and host cell closer, and help VP28 to attach the host cell and the virus enter the cytoplasm.

Based on our work combined with previously studies, it is suggested that VP24, VP26 and VP28 all favor trimers with a weak interaction when aggregated in the envelope and could adopt different oligomerization states to exhibit different functions. While VP26 may locate inside viral envelop, VP24 and VP28 may sit outside viral envelope with VP24 barrel attaching more closely to the envelope (Fig. 7). Through the extended dynamic C-terminus, VP24 may interact with more structural protein partners for WSSV assembly or infection. Furthermore, VP24 may bind to chitin of host cell via strand β9, anchoring WSSV to the host cell and facilitating the attachment of VP28 to the host cell and membrane fusion, and initiate virus infection. Hence, our structure will contribute to an in-depth investigation and understanding of the molecular mechanism of WSSV assembly and infection, and in turn provide valuable information on neutralizing antibody design and vaccine development against WSSV.

## Materials and Methods

### Cloning, expression, and purification

The gene coding for the truncated constructs of VP24 was amplified from the WSSV genome and inserted into vector pET21b, by using restriction enzymes NdeI and XhoI, resulting in a C-terminal hexahistidine tag for purification. Sequence analysis showed that only one Met site was presented in truncated VP24 sequence. So some mutants (L69M, L88M, L121M, I133M, and L69M/L121M) were constructed to increase the ratio of Met. The mutants were generated by site-directed mutagenesis using the pET21b-VP24 vector as the template and confirmed by sequencing. All recombinant plasmids were transformed into *Escherichia coli* BL21 (DE3). Cells were grown to an OD600 of 0.8 at 37 °C, and VP24-His6 fusion protein expression was induced by 0.3 mM isopropyl-β-D-thiogalactoside (IPTG) overnight at 16 °C. Cells were centrifuged, resuspended in lysis buffer containing 25 mM Tris-HCl (pH 7.0), 300 mM NaCl, 5% glycerol and sonicated on ice. Cell debris was removed by centrifugation. Purification was achieved by Ni-NTA affinity chromatography (GE Healthcare), gel filtration (Superdex 200 HR 16/60; GE Healthcare). Mutants were measured by Circular dichrosim spectra to estimate the stability of their secondary structure. Seleno-methinonine (SeMet, Sigma-Aldrich) labeling of mutants (L69M and L69M/L121M) were produced by inhibiting endogenous methionine biosynthesis in M9 minimal medium supplemented with specific amino acids as well as SeMet and then purified in the similar protocol as the native protein^42^,^43^. Purified protein fractions were collected and concentrated to a final concentration of 2.5 to 3.5 mg/ml plus with 0.5 M Non Detergent Sulfobetanines NDSB-201 (Sigma).

### Crystallization and data collection

Crystals of VP24 and mutants (L69M, L69M/L121M) were obtained using the sitting drop method at 16 °C. The initial screens were carried out using Qiagen crystallization screen kits (the JCSG Core I-IV suites). The initial conditions were further optimized to obtain diffraction-quality crystals. The best crystals of VP24 were found with a reservoir solution of 0.1M Tris-HCl, pH 8.5, 2.75 M Ammonium acetate with drop volume ratio of two parts protein: one parts reservoir solution by using the hanging-drop vapor diffusion method. 10 mM ATP was as a critical additive in present of the drop. Prior to data collection, crystals were briefly soaked in a cryo-protectant solution consisting of 30% glycerol, picked up in a CryoLoop, and flash-cooled at liquid nitrogen. The X-ray diffraction data sets were collected for single anomalous diffraction (SAD) phasing using the beamline BL17U at Shanghai Synchrotron Radiation Facility (SSRF, shanghai, China) using a charge-coupled device (CCD) detector.

### Structure determination, and refinement

Data sets were integrated and scaled using HKL2000 package. Further processing was carried out using programs from the CCP4 suite^44^. Phasing was achieved by SAD method by using L69M/L121M-Se data set. SHELXD was used to locate the positions of selenium sites^45^. After phase calculation, phase extension, and phase improvement by density modification, the initial model was built automatically by Phenix program AutoSol containing about 80% of the polypeptide (~amino acids) for VP24. Then native VP24 was determined by the molecular replacement, using the initial model as the search model and the native dataset to refine. Iterative cycles of manual rebuilding and maximum likelihood refinement were performed by Coot^46^ and Phenix^47^. All structure figures were prepared by using PyMOL program (DeLano Scientific LLC). Sequence alignment was generated with DaliLite server^48^. The atomic coordination and structure factors for VP24 have been deposited in the Protein Data Bank under the accession code of 5HLJ. The data collection and refinement statistics were listed in Table 1.

### Cross-linking assay

For nonspecific cross-linking, purified protein in PBS buffer (around 0.6 mg/ml in 40 ul) was incubated with various concentrations of glutaraldehyde (0, 0.0001%, 0.001%, 0.01%) at room temperature for 4 h, respectively. The reaction was quenched by addition of 50 mM Tris-HCl, pH 8.0. The sample was mixed with equal volume of SDS loading buffer, separated on 15% SDS-PAGE gels and stained with Coomassie blue.

### Isothermal titration calorimetry (ITC) assays

The dissociation constant (Kd) and stoichiometry of the interaction between VP24 and VP28 or VP26 were measured by ITC using an ITC200 calorimeter (GE Healthcare). Calorimetric titration of VP28 (0.3 mM in the syringe; 2 μl injections) or VP26 (0.2 mM in the syringe; 2 μl injections) to VP24 (0.012 mM or 0.03 mM in the cell, 200 μl) was performed at 25 °C in assay buffer containing 25 mM Tris-HCl, pH7.0, 200 mM NaCl, 5% glycerol or buffer containing 25 mM Tris-HCl, pH7.0, 100 mM NaCl. Time between injections was 150 s. ITC data were analyzed by integrating the heat effects after the data were normalized to the amount of injected protein. Data fitting was conducted to determine the dissociation constant and stoichiometry based on a single-site binding model using the Origin software package (MicroCal).

## Additional Information

**Accession codes:** The crystal structure of VP24 has been submitted to the Protein Data Bank with the 397 accession code of 5HLJ.

**How to cite this article**: Sun, L. *et al.* Crystal Structure of Major Envelope Protein VP24 from White Spot Syndrome Virus. *Sci. Rep.* **6**, 32309; doi: 10.1038/srep32309 (2016).

## Supplementary Material

Supplementary Information

## Figures and Tables

**Table 1:** Data collection and refinement statistics of VP24.

Data collection	VP24	L69M/L121M-Se
Space group	I 2_1_ 3	I 2_1_ 3
Cell dimensions		
a, b, c (Å)	140, 140, 140	140, 140, 140
α, β, γ (°)	90, 90, 90	90, 90, 90
Resolution (Å)	2.40–50 (2.40–2.44)	2.8–44 (2.80–2.85)
R_merge_ (%)	7.6 (94)	10 (77.6)
I/σI	51 (1.65)	32.6 (3.04)
CC1/2	(0.663)	(0.729)
Completeness (%)	99.83 (100)	98.53 (100)
Redundancy	20.6 (20.4)	7.3 (7.5)
Wilson B-factor (Å^2^)	71.49	72.74
Refinement		
Resolution (Å)	2.4–31.25	
No. reflections	17728	
R_work_/R_free_ (%)	17.08/19.26	
No. atoms	1382	
Water	54	
B factors		
Protein	71.86	
Water	74.85	
R.m.s.d bonds (Å)	0.008	
R.m.s.d angles (°)	1.125	
Ramachandran plot		
Favored (%)	96.41	
Allowed (%)	3.59	
Outliers (%)	0.00	
Rotamer outliers (%)	0.00	

Numbers in parentheses refer to the highest-resolution shell.
