# Supplementary material for: Crystal Structure of Major Envelope Protein VP24 from White Spot Syndrome Virus
Source: Sci Rep. 2016 Aug 30;6:32309. doi: 10.1038/srep32309 (PMC5004148; doi:10.1038/srep32309)
Supplement: Supplementary Information [file srep32309-s1.pdf]

Supplemental figure

Crystal Structure of Major Envelope Protein VP24 from White Spot Syndrome Virus

Lifang Sun[a], Yintao Su[a], Yanhe Zhao[a], Zheng-qing Fu[b], Yunkun Wu[a*]

State Key Laboratory of Structural Chemistry, Fujian Institute of Research on the Structure of Matter, Chinese Academy of Sciences, Fuzhou 350002, China[a];

Department of Biochemistry and Molecular Biology, University of Georgia, Athens, GA 30602, USA[b]

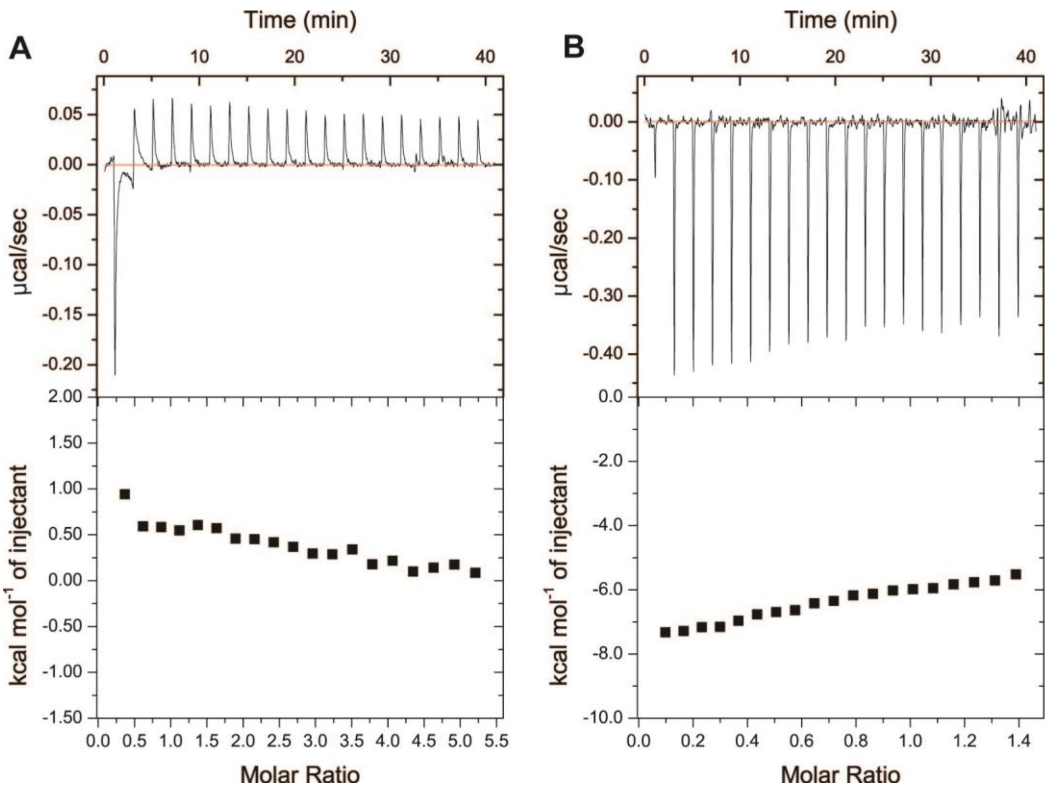

Supplementary Figure. Analysis the interaction between VP24 and VP28 (A), VP24 and VP26 (B) by ITC assay.
